# Cancer Labeling, Risk Perception, and Treatment Choices in Clonal Cytopenia of Undetermined Significance

**DOI:** 10.1001/jamanetworkopen.2025.23733

**Published:** 2025-07-29

**Authors:** Benjamin Chin-Yee, Andrew J. Latham, Somogy Varga

**Affiliations:** 1 Department of Pathology and Laboratory Medicine, Department of Medicine, and Department of Philosophy, Western University, London, Ontario, Canada; 2 Department of Pathology and Laboratory Medicine, London Health Sciences Centre, London, Ontario, Canada; 3 Department of History and Philosophy of Science, University of Cambridge, Cambridge, United Kingdom; 4 Department of Philosophy and History of Ideas, Aarhus University, Aarhus, Denmark; 5 Centre for Philosophy of Epidemiology, Medicine and Public Health, University of Johannesburg, Johannesburg, South Africa

## Abstract

This survey study investigates whether diagnostic labels and linguistic framing are associated with risk perceptions and treatment decisions for patients with clonal cytopenia of undetermined significance (CCUS).

## Introduction

With increasing detection of early cancers and precancers, debate over the cancer label has intensified.^1^ Although attention focuses on relabeling solid tumors to reduce overtreatment, hematologic precursor conditions remain overlooked. Clonal cytopenia of undetermined significance (CCUS) carries increased risk of progression to myelodysplastic neoplasms and acute myeloid leukemia, with high-risk CCUS having a prognosis similar to that of lower-risk myelodysplastic neoplasms.^2^ Because these conditions are managed with surveillance or systemic therapy based on risk, diagnostic labels and linguistic framing may influence decision-making. We investigated whether these factors are associated with changes in risk perceptions and treatment decisions in CCUS.

## Methods

We conducted a vignette-based survey study from February 6 to 13, 2025, with US and UK participants, recruited through Prolific, randomly assigned to 1 of 32 unique vignettes generated using a factorial design. These described a patient with CCUS, varying by labeling as “early blood cancer” or “blood disorder”; attribution to “genetic mutation” or “aging”; describing treatment as “precision” or “standard”; using battle metaphors (“fight the disease”) or neutral language; and third-person (“Alex”) or second-person (“you”) framing. Participants rated (7-point Likert scale) perceived seriousness of the condition, treatment benefit, and choice of treatment. Separate analyses of variance were performed for each dependent variable with a preregistered significance level of *P* = .005. Data were analyzed with SPSS statistical software version 29 (IBM). The study was preregistered on Open Science Framework; followed AAPOR best practices for design, analysis, and reporting; and received approval from the Aarhus University Human Ethics Committee (eMethods in Supplement 1). Participants provided consent through the return of a completed survey.

## Results

The response rate was 97% (802 of 830 participants); 400 UK participants (196 female [49%]; mean [SD] age, 39.54 [13.93] years) and 402 US participants (194 female [48%]; mean [SD] age, 39.73 [12.97] years) completed the survey (eMethods in Supplement 1). Labeling CCUS as cancer was associated with increased perceptions of seriousness among US (cancer, mean [SE] score, 4.16 [0.12]; blood disorder, mean [SE] score, 3.47 [0.12]; difference, 0.69; 95% CI, 0.35-1.03) and UK (cancer, mean [SE] score, 4.33 [0.12]; blood disorder, mean [SE] score, 3.45 [0.12]; difference, 0.88; 95% CI, 0.56-1.20) participants (Table). It also was associated with increased perceptions of treatment benefit among US (cancer, mean [SE] score, 3.78 [0.12]; blood disorder, mean [SE] score, 3.19 [0.12]; difference, 0.59; 95% CI, 0.25-0.92) and UK (cancer, mean [SE] score, 3.94 [0.12]; blood disorder, mean [SE] score, 3.37 [0.12]; difference, 0.57; 95% CI, 0.25-0.89) participants. Labeling CCUS as cancer was associated with changes in treatment choice among UK participants only (cancer, mean [SE] score, 4.08 [0.12]; blood disorder, mean [SE] score, 3.25 [0.12]; difference, 0.83; 95% CI, 0.48-1.16). Genetic attribution, precision medicine framing, and battle metaphors were not independently associated with changes in participants’ judgments. UK participants rated CCUS as more serious when described in second person vs third person (mean [SE] score, 4.18 [0.12] vs 3.60 [0.12]; difference, 0.58; 95% CI, 0.26-0.90).

**Table.** Perceptions of Condition Seriousness, Treatment Benefit, and Treatment Choice by Diagnostic Labeling and Linguistic Framing Factors^a^

Labeling	Condition seriousness				Treatment benefit				Choice of treatment			
	US		UK		US		UK		US		UK	
	*F*_1,366_	*P* value	*F*_1,366_	*P* value	*F*_1,366_	*P* value	*F*_1,366_	*P* value	*F*_1,366_	*P* value	*F*_1,366_	*P* value
Cancer vs blood disorder	16.156	<.001^b^	28.763	<.001^b^	11.533	<.001^b^	12.036	<.001^b^	3.491	.06	22.270	<.001^b^
Genetic mutation vs aging	2.077	.15	0.309	.58	0.018	.89	0.073	.79	0.014	.91	0.210	.65
Precision vs standard treatment	0.069	.79	0.561	.45	0.005	.95	3.837	.05	0.013	.91	0.133	.72
Battle metaphor vs neutral language	0.099	.75	0.157	.69	0.814	.37	0.672	.41	0.822	.37	0.466	.50
Third vs second person	3.565	.06	12.430	<.001^b^	0.839	.36	4.171	.04	4.597	.03	2.428	.12

^a^ Table shows main outcomes associated with the following factors: labeling as “early blood cancer” vs “blood disorder,” attribution to “genetic mutation” vs “aging,” describing treatment as “precision” or “standard,” using battle metaphors (“fight the disease”) vs neutral language, and third-person (“Alex”) vs second-person (“you”) framing on US and UK participants’ judgments of condition seriousness, treatment benefit, and decision to undergo treatment. Condition seriousness was assessed by examining levels of agreement to the statement, “In this scenario, I believe that Alex/I have a serious, potentially life-threatening condition.” Treatment benefit was assessed by examining levels of agreement to the statement, “In this scenario, I believe that the benefits of immediate treatment outweigh the risks of watchful waiting.” Finally, choice of treatment was assessed by examining levels of agreement to the statement, “In this scenario, I believe that Alex should/I would choose immediate treatment.”

^b^ Indicates a statistically significant association according to the preregistered *P* = .005.

## Discussion

This survey study found that labeling alone can shift risk perception and treatment choices in CCUS. Although the cancer label amplified perceived risk and treatment benefit, it was not associated with changes in treatment decisions among US participants. This contrasts with prior research on solid tumors,^3,4,5^ where relabeling was associated with reduced preference for surgery. One possible explanation is that surgery carries familiar risks, discouraging overtreatment of indolent conditions. In contrast, hematological conditions are typically managed nonsurgically, which may seem less risky, leading to higher treatment preference regardless of labeling. Interestingly, the cancer label was associated with an increased preference for treatment among UK participants, suggesting that additional factors—such as health care access or costs—may also affect decision-making.

Linguistic framing was not independently associated with changes in participants’ responses. The lack of effect from battle metaphors, although theorized to reinforce need for intervention,^6^ suggests they may rely on broader societal discourse and prior exposure rather than brief encounters in experimental settings. Limitations include the vignette-based design, which cannot replicate real-world clinical decision-making, recruitment from an online platform, which may bias toward more tech-literate users, focus on a single hematologic condition, and potential insensitivity to subtle linguistic effects.

This study, which to our knowledge is the first to examine labeling effects in CCUS, supports evidence that cancer labeling may influence judgments about risk and treatment, but highlights how effects vary by context and disease type. As debates continue,^1^ our findings underscore the need to consider not just terminology but also its clinical and communicative context.

## Notes

Supplement 1.
eMethods.

Supplement 2.
Data Sharing Statement
