# Supplementary material for: Cancer Labeling, Risk Perception, and Treatment Choices in Clonal Cytopenia of Undetermined Significance
Source: JAMA Netw Open. 2025 Jul 29;8(7):e2523733. doi: 10.1001/jamanetworkopen.2025.23733 (PMC12308432; doi:10.1001/jamanetworkopen.2025.23733)
Supplement: Supplement 1. — eMethods. [file jamanetwopen-e2523733-s001.pdf]

Supplemental Online Content

Chin-Yee B, Latham AJ, Varga S. Cancer labeling, risk perception, and treatment choices in clonal cytopenia of undetermined significance. *JAMA Netw Open*. 2025;8(7):e2523733. doi:10.1001/jamanetworkopen.2025.23733

eMethods.

This supplemental material has been provided by the authors to give readers additional information about their work.

© 2025 Chin-Yee B et al. *JAMA Network Open*.

eMethods

Experimental Design

This study tested the impact five factors on participants' judgements using a 2x2x2x2x2 factorial design, randomly assigning participants to one of 32 unique vignettes. These factors were selected based on theoretical considerations from existing literature and empirical evidence for influence on patient-decision making. The first factor (*cancer*) manipulated whether the word "cancer" was used to describe the condition. The second factor (*mutation*) manipulated whether the condition was described as associated with a genetic mutation. The third factor (*precision*) manipulated whether the treatment was described using the language of "precision medicine." The fourth factor (*metaphor*) manipulated whether management of the condition was described using bellicose language, namely battle metaphors. The fifth factor (*person*) manipulated whether management of the condition was described in second or third-person.

Vignettes

The development of the vignettes involved an iterative process informed by theoretical considerations, prior empirical literature, and expert feedback. The five factors were selected based on existing literature and relevance to patient decision-making:

1. Labeling a condition as "early blood cancer" vs. "blood disorder": Disease labels, particularly the use of the term "cancer," have been shown to affect perceptions of seriousness and treatment preferences in the context of solid tumors (e.g., Omer et al. *JAMA Intern Med* 2013; Nickel et al. *JAMA Otolaryngol Head Neck Surg* 2018; Dixon et al. *JAMA Oncol* 2019; Berlin et al. *J Natl Cancer Inst* 2023).
2. Attribution to genetic mutation vs. aging: Explanations based on genetics or aging can influence beliefs about controllability, personal responsibility, and treatment effectiveness (e.g., Stewart et al. *Psychol Health* 2012; Wright et al. *Br J Health Psychol* 2012)
3. Describing treatment as "precision" vs. "standard": Framing therapies as "precision medicine" may lead to more favorable perceptions of effectiveness and appeal (e.g., Erdmann et al. *BMC Med Ethics* 2021; Kim et al. *Front Genet* 2020).
4. Use of battle metaphors vs. neutral language: Metaphoric language, such as battle/war metaphors, has been shown to influence emotional responses, motivation, and decision-making, though the effects may vary by context and individual (e.g., Hauser & Schwarz *Health Commun* 2020; Bodd et al. *Support Care Cancer* 2023).
5. Second person ("you") vs. third-person ("Alex") framing: Prior work suggests that decision-making may differ depending on whether individuals are imagining themselves or others in a scenario, potentially due to emotional salience or perceived responsibility (e.g., Ubel et al. *Arch Intern Med* 2011; Zikmund-Fisher et al. *J Gen Intern Med* 2006).

© 2025 Chin-Yee B et al. *JAMA Network Open*.

We created draft versions of the vignettes using these factors. These drafts were reviewed by colleagues specializing in clinical oncology/hematology and philosophy/psychology. The experts were asked to evaluate the clarity, accuracy, and potential biases in the language used. Feedback was gathered through structured discussions and written comments, which informed subsequent revisions. This process ensured that the final vignettes were both conceptually grounded and presented in a manner consistent with clinical communication practices.

<u>Third-Person Scenario</u>

Alex, a 65-year-old retiree, is referred to a doctor because of abnormal blood counts that were found at a routine check-up. Alex feels fine—they have no symptoms, and all other tests were normal. After evaluation, the doctor diagnoses Alex with a condition called "Clonal Cytopenias of Undetermined Significance (CCUS)."

The doctor explains that CCUS is **[an early form of blood cancer / a blood condition]** with a low risk of progression to bone marrow failure and a small risk of death. The cause of the condition is uncertain but is **[associated with a genetic mutation in Alex's blood cells / associated with aging]**.

There are two options for management. The first is watchful waiting to see if the condition progresses. The second is immediate treatment. The doctor describes the first-line treatment as **[a precision drug tailored to Alex's condition / a standard treatment used for Alex's condition]**. The treatment offers a small chance of curing the condition, but it also comes with a small risk of death.

The doctor provides Alex with all the relevant information about their condition and the treatment but emphasizes that the decision to undergo treatment is entirely theirs to make. The doctor says to Alex: "Some patients choose **[to treat CCUS and are cured / to fight CCUS head-on and defeat it, achieving a cure]**; others choose to live with the condition. Both approaches are reasonable."

<u>Second Person Scenario</u>

You are referred to a doctor because of abnormal blood counts that were found at a routine check-up. You feel fine—you have no symptoms, and all other tests were normal. After further evaluation, the doctor diagnoses you with a condition called "Clonal Cytopenias of Undetermined Significance (CCUS)."

The doctor explains that CCUS is **[an early form of blood cancer / a blood condition]** with a low risk of progression to bone marrow failure and a small risk of death. The cause of the condition is uncertain but is **[associated with a genetic mutation in your blood cells / associated with aging]**.

© 2025 Chin-Yee B et al. *JAMA Network Open*.

There are two options for management. The first is watchful waiting to see if the condition progresses. The second is immediate treatment. The doctor describes the first-line treatment as **[a precision drug tailored to your condition / a standard treatment used for your condition]**. The treatment offers a small chance of curing the condition, but it also comes with a small risk of death.

The doctor provides you with all the relevant information about your condition and the treatment but emphasizes that the decision to undergo treatment is entirely yours to make. The doctor says: "Some patients choose **[to treat CCUS and are cured / to fight CCUS head-on and defeat it, achieving a cure]**; others choose to live with the condition. Both approaches are reasonable."

Probe Questions

Third-Person Scenario Probe Questions

Probe questions asked participants their level of agreement with each statement on a seven-point Likert scale, ranging from "strongly disagree" to "strongly agree."

Q1. In this scenario, I believe that Alex has a serious, potentially life-threatening condition.

Q2. In this scenario, I believe that the doctor thinks immediate treatment would be best for Alex.

Q3. In this scenario, I believe Alex is healthy.

Q4. In this scenario, I believe that the doctor thinks that the benefits of immediate treatment outweigh the risks of watchful waiting.

Q5. In this scenario, I believe that the benefits of immediate treatment outweigh the risks of watchful waiting.

Q6. In this scenario, I believe that Alex should choose to undergo immediate treatment.

Second Person Scenario Probe Questions

Probe questions asked participants their level of agreement with each statement on a seven-point Likert scale, ranging from "strongly disagree" to "strongly agree."

Q1. In this scenario, I believe that I have a serious, potentially life-threatening condition.

Q2. In this scenario, I believe that the doctor thinks immediate treatment would be best for Alex.

Q3. In this scenario, I am healthy.

Q4. In this scenario, I believe that the doctor thinks that the benefits of immediate treatment outweigh the risks of watchful waiting.

Q5. In this scenario, I believe that the benefits of immediate treatment outweigh the risks of watchful waiting.

© 2025 Chin-Yee B et al. *JAMA Network Open*.

Q6. In this scenario, I would choose immediate treatment.

Recruitment

Participants were recruited through Prolific (https://www.prolific.com/), a widely used online research platform known for its diverse and vetted participant pool. Prolific ensures data quality by verifying participant identities and employing robust quality control measures, including attention checks and monitoring of response consistency.

For this study, we applied demographic filters to recruit adult participants residing in the US and UK, ensuring they were aged 18 years or older and fluent in English. Prolific's automated randomization tool was used to assign participants to experimental conditions.

Compensation was provided in accordance with Prolific's guidelines, which was $2 USD for approximately 10 minutes of time in this study. Prolific's recruitment process, while rigorous and diverse, may introduce biases related to demographic characteristics and technology literacy.

Sample Size

We conducted a power analysis before data collection to determine the appropriate sample size. Based on our goal of obtaining 0.9 power to detect a medium effect size at 0.005 alpha error probability (Benjamin et al. *Nat Hum Behav* 2018), we calculated that a minimum of 272 participants would be required. To account for attention check and comprehension failures, we increased this by 45%, resulting in a sample size of 395 participants per country, which we rounded up to 400. See eTables for further details on US (https://osf.io/zbyqu) and UK (https://osf.io/nh9vb) samples.

Statistical Analysis

Statistical analysis was performed with separate 2x2x2x2x2 ANOVAs for each dependent variable. Interaction effects were examined using a Bonferroni correction. A significance level of 0.005 was used for this study. All hypotheses and methods were preregistered prior to conducting the study and are publicly available through OSF (US: https://osf.io/tp2zn; UK: https://osf.io/sev3r).

Study Dates

The survey was administered between February 6 and February 13, 2025.

Survey Quality and Reporting Standards

This study was preregistered on Open Science Framework and followed American Association for Public Opinion Research (AAPOR) best practices for survey research (https://aapor.org/standards-and-ethics/best-practices/). In line with these recommendations, we clearly defined our study objectives, described recruitment and sampling procedures, endeavoured to use neutrally worded survey items, and employed random assignment to experimental conditions. All methods and

© 2025 Chin-Yee B et al. *JAMA Network Open*.

materials were preregistered and made openly available through OSF, in keeping with AAPOR's emphasis on transparency and reproducibility.

© 2025 Chin-Yee B et al. *JAMA Network Open*.
