# Supplementary material for: Cancer Labeling, Risk Perception, and Treatment Choices in Clonal Cytopenia of Undetermined Significance
Source: JAMA Netw Open. 2025 Jul 29;8(7):e2523733. doi: 10.1001/jamanetworkopen.2025.23733 (PMC12308432; doi:10.1001/jamanetworkopen.2025.23733)
Supplement: Supplement 2. — Data Sharing Statement [file jamanetwopen-e2523733-s002.pdf]

# Data Sharing Statement

Chin-Yee. Cancer Labeling, Risk Perception, and Treatment Choices in Clonal Cytopenia of Undetermined Significance. *JAMA Netw Open*. Published July 29, 2025. doi:10.1001/jamanetworkopen.2025.23733

**Data**
**Data available:** Yes
**Data types:** Deidentified participant data
**How to access data:** Data will be made available through the pre-registration on Open Science Framework (US: https://osf.io/e4c5a/; UK: https://osf.io/pgv2c/).
**When available:** With publication

**Supporting Documents**
**Document types:** None

**Additional Information**
**Who can access the data:** Data will be made available to all through the pre-registration on Open Science Framework.
**Types of analyses:** Data will be made available to all through the pre-registration on Open Science Framework.
**Mechanisms of data availability:** Data will be made available to all through the pre-registration on Open Science Framework.
